# MHC class I diversity of olive baboons (*Papio anubis*) unravelled by next-generation sequencing

**DOI:** 10.1007/s00251-018-1053-7

**Published:** 2018-02-24

**Authors:** Marit K. H. van der Wiel, Gaby G. M. Doxiadis, N. de Groot, N. Otting, N. G. de Groot, N. Poirier, G. Blancho, R. E. Bontrop

**Affiliations:** 1 Department of Comparative Genetics and Refinement, Biomedical Primate Research Centre, Lange Kleiweg 161, 2288 GJ Rijswijk, The Netherlands; 2 Institut de Transplantation Urologie-Nephrologie (ITUN), UMR1064, Institut National de la Sante et de la Recherche Medicale (INSERM), 30 Bd Jean Monnet, 44093 Nantes, France; 3 Department of Theoretical Biology and Bioinformatics, Utrecht University, Padualaan 8, 3584 CH Utrecht, The Netherlands

**Keywords:** MHC, Next-generation sequencing, Non-human primates, Haplotyping

## Abstract

The olive baboon represents an important model system to study various aspects of human biology and health, including the origin and diversity of the major histocompatibility complex. After screening of a group of related animals for polymorphisms associated with a well-defined microsatellite marker, subsequent MHC class I typing of a selected population of 24 animals was performed on two distinct next-generation sequencing (NGS) platforms. A substantial number of 21 *A* and 80 *B* transcripts were discovered, about half of which had not been previously reported. Per animal, from one to four highly transcribed *A* alleles (majors) were observed, in addition to ones characterised by low transcripion levels (minors), such as members of the A*14 lineage. Furthermore, in one animal, up to 13 *B* alleles with differential transcription level profiles may be present. Based on segregation profiles, 16 *Paan-AB* haplotypes were defined. A haplotype encodes in general one or two major *A* and three to seven *B* transcripts, respectively. A further peculiarity is the presence of at least one copy of a B*02 lineage on nearly every haplotype, which indicates that B*02 represents a separate locus with probably a specialistic function. Haplotypes appear to be generated by recombination-like events, and the breakpoints map not only between the *A* and *B* regions but also within the *B* region itself. Therefore, the genetic makeup of the olive baboon MHC class I region appears to have been subject to a similar or even more complex expansion process than the one documented for macaque species.

**Electronic supplementary material:**

The online version of this article (10.1007/s00251-018-1053-7) contains supplementary material, which is available to authorized users.

## Introduction

The olive baboon (*Papio anubis*), an Old World monkey (OWM) species, has its natural habitat in equatorial Africa, and it often serves as a model species in immune response-related studies such as in renal and xeno-transplantation research, as well as in immunotoxicity and sepsis protocols (Le Bas-Bernardet et al. 2015; Poirier et al. 2014, 2015; Schochl et al. 2017; Tanabe et al. 2017). Since the major histocompatibility complex (MHC) plays an important role in generating adaptive immune responses, characterisation of the polymorphic genes encoding MHC class I and II allotypes, which are peptide receptors, is necessary. The *DR* region of this species has recently been characterised, and a high level of copy number variation (CNV) was observed, allowing the definition of 19 haplotypes. The number of *DRB* genes present per chromosome varies from two to five. As found in macaques, the *DRB* region (*Paan-DRB*) in the olive baboon shows a high level of region configuration polymorphism, whereas allelic variation appears to be absent (de Groot et al. 2017a). Furthermore, the *Paan*-*DQ*, *Paan*-*DP*, and *Paan*-*DRA* genes have been analysed by full-length cDNA sequencing, demonstrating substantial levels of allelic heterogeneity (Otting et al. 2016).

Little is known about the olive baboon MHC class I genes. We wished to investigate whether the *Paan-A* and *Paan-B* genes exhibit copy number variation (CNV) and/or allelic polymorphism, potentially in concert with transcription level differences, as has been documented for other OWM. In rhesus and cynomolgus monkeys, for example the *A* genes are duplicated, and at the population level, one to eight *A* (*A1*–*A8*) loci can be defined. At the haplotype level, generally one major *A1* gene is present, which is highly polymorphic, in combination with a selected set of one to three minor *A* genes (*A2*–*A7*) that are oligomorphic and display low transcription levels. The *Mamu-A2* gene was recently reported to be a specialist, which preferentially presents 8mer peptides (de Groot et al. 2017b). The *B* region in macaques shows even more CNV, and up to seven major and eight minor genes may be defined per haplotype. In contrast to the *A* region, however, a clear differentiation between major and minor *B* genes is difficult, as well as a definitive allocation of alleles to a certain locus (Budde et al. 2010; Doxiadis et al. 2011, 2013; Fernandez et al. 2011; Karl et al. 2013, 2017; Naruse et al. 2010; O’Leary et al. 2009; Otting et al. 2005, 2007, 2009, 2012; Saito et al. 2012; Shiina et al. 2015).

In different macaque species, two microsatellites—namely, D6S2854 and D6S2859—have been proven to display length pattern polymorphism and can be used to discriminate between macaque *A* haplotypes (Doxiadis et al. 2011). Therefore, we first screened the olive baboon cohort (*N* = 154) with these two microsatellites. Since these pre-screening results had already indicated the potentially high level of CNV, a combination of different techniques and platforms was used to shed light on the MHC class I transcriptome of olive baboons. The results are compared to the information that is available for thoroughly characterised macaque species.

## Material and methods

### Animals

Olive baboons, originally obtained from the Centre National de la Recherche Scientifique Centre de Primatologie (Rousset, France), were housed at the large animal facility of the INSERM unit 1064 (Nantes, France). The baboons belong to a breeding group of 154 animals, which are pedigreed and descended from 34 sires and 105 dames. In most cases, the sires were readily identified; based on genetic similarities, however, sometimes two males could potentially have sired the offspring (de Groot et al. 2017a). The 24 animals analysed in this study for class I cDNA with NGS were part of this group.

### DNA extraction

Blood sampling of the 154 related olive baboons, belonging to the cohort described above, was performed under anaesthesia at the facilities of INSERM, Nantes, in accordance with the institutional ethical guidelines. DNA was extracted from EDTA or citrate blood samples of the 154 animals as described recently, using a conventional phenol/chloroform method (de Groot et al. 2017a).

### *Paan-A* D6S2854 and D6S2859 microsatellite typing

Primers and conditions for the PCR amplification of STR markers D6S2854 and D6S2859 have previously been described (Doxiadis et al. 2011; Wiseman et al. 2007). Briefly, PCR reactions were multiplexed in a 25-μl reaction volume containing 1 unit of *Taq* polymerase (Invitrogen, Paisley, Scotland) with 0.3 μM of the forward and reverse primer of D6S2859, 0.1 μM of the forward and reverse primer of D6S2854, 5 mM MgCl_2_, 0.2 mM of each dNTP, 1× PCR buffer II (Invitrogen, Paisley, Scotland), and 100 ng DNA. The cycling parameters were a 5-min 94 °C initial denaturation step, followed by 5 cycles of 1 min at 94 °C, 45 s at 58 °C and 45 s at 72 °C. The programme was followed by 25 cycles of 45 s at 94 °C, 30 s at 58 °C and 45 s at 72 °C. A final extension step was performed at 72 °C for 30 min. The amplified DNA was prepared for genotyping and analysed on an ABI 3130*XL* genetic analyser (Applied Biosystems). STR analysis was performed using the Genemapper software (Applied Biosystems).

### RNA isolation and cDNA synthesis

Blood sampling of 24 related olive baboons, belonging to the cohort described above, was performed under anaesthesia at the facilities of INSERM, Nantes, in accordance with the institutional ethical guidelines. Peripheral blood mononuclear cells (PBMC) were isolated from whole blood by density centrifugation over Ficoll-Paque (Eurobio, Courtaboeuf, France), and sent to BPRC on dry ice. RNA was isolated at BPRC using the All prep DNA/RNA mini kit (QIAGEN Benelux B.V) following the manufacturer’s instructions.

cDNA was produced from the isolated RNA using the RevertAid First Strand cDNA Kit (ThermoFisher Scientific) in accordance the manufacturer’s instructions.

### Roche 454 sequencing

#### PCR amplification

For Roche 454 Junior genotyping, using the long-range sequencing GS Junior + series-XL kit, a 755-bp amplicon was produced spanning the polymorphic exon 2 and 3 regions of MHC class I. For the amplification of *Paan-AB* in 24 olive baboons, 48 MID primers (reactions *in duplo*) and one barcode (BC) were used for two subsequent PCR reactions. The initial BC-PCR comprising a forward/reverse adaptor sequence, a barcode sequence, and a target-specific, newly defined sequence was performed with a few cycles to reduce any preferential amplification of specific alleles (Suppl. Table 1). The initial PCR was performed in a volume of 20 μl with 0.2 mM dNTPs, 0.5 μM each of the forward and reverse BC primers, 3% DMSO, 1× EVAgreen (Biotium, Hayward, US), 5 μl cDNA, and 0.02 U/μl Phusion polymerase (Phusion Hot Start II High Fidelity Polymerase, Finnzymes, Vantaa, Finland) in 1× Phusion HF buffer. The cycle conditions were a hot-start of 1 min at 98 °C, followed by 10 cycles of 15 s at 98 °C, 10 s at 66 °C, 10 s at 63 °C, 10 s at 60 °C, 30 s at 58 °C and 30 s at 72 °C, with a final extension of 40 s 72 °C.

Any non-extended oligos and single-stranded DNA were then enzymatically digested by exonuclease I (Thermo Scientific, Leon-Rot, Germany) by adding 1 μl of 5× Phusion HF buffer, 3.5 μl H_2_O and 0.5 μl exonuclease I (10 units) to the PCR product. Digestion was performed for 30 min at 37 °C, and the enzyme was then inactivated for 15 min at 80 °C.

For the second, quantitative PCR, 5 μl of a reaction mix containing 1× Phusion HF buffer, 0.2 μM dNTPs, 0.02 U/μl Phusion HS II HF Polymerase and 0.5 μM each of forward and reverse MID primers (Suppl. Table 1) was added to the exonuclease-treated, first PCR product. Cycling conditions were a hot-start of 1 min at 98 °C, followed by 20 cycles of 10 s at 98 °C, 20 s at 68 °C and 30 s at 72 °C, with a final extension of 1 min at 72 °C.

#### Library preparation

For each sample, the end point fluorescence was determined, and amplification was considered successful if 100 relative fluorescence units (RFU) was exceeded. All reactions were divided into groups of four based on similar end point RFU, and from these groups, a four times 15 μl reaction mix was pooled and run on a 1.5% agarose gel (12 samples were not pooled). The band that corresponded to a size of 755 bp for *Paan-AB* was excised and purified by a GeneJET gel extraction kit (Thermo Scientific, Leon-Rot, Germany). Finally, the amplicons were eluted with 50 μl elution buffer, and the concentration of 5 μl from each pool was measured with a Qubit fluorometer (Life technologies, Paisley, UK). Based on these measurements, all samples were pooled equimolar, followed by a further concentration measurement and a final dilution of pooled products to 2 million molecules/μl. This final mix was used for emulsion PCR.

#### Emulsion PCR and Roche 454 sequencing

Emulsion PCR was performed according to the emPCR Amplification Method Manual—LIB-A of Roche GS Junior+ Series (Roche, Mannheim, Germany). Preparation of samples for the Roche 454 sequencing was also performed according to the Sequencing Manual—LIB-A of Roche GS Junior+ Series protocol (Roche, Mannheim, Germany), and long-range sequencing was run on Roche 454 Junior or FLX instruments. An average of 450 MHC class I reads was obtained for each animal.

#### Data analysis

Raw data generated by Roche 454 Junior/FLX were first analysed in Roche 454 Analysis software version 2.6 to get rid of NGS-dependent sequencing errors; fna files were then imported and analysed in Geneious Pro (version 9.0) (Biomatters Limited, Auckland, New Zealand). All sequences were first compared to the NCBI (https://blast.ncbi.nlm.nih.gov/Blast.cgi) and the IPD-MHC NHP databases (http://www.ebi.ac.uk/ipd/mhc/group/NHP) to define known alleles. All other sequences were de novo assembled and compared to each other. Only those that had been detected in forward and reverse directions and at least ten times with zero mismatches were accepted as new alleles.

### PacBio full-length sequencing

The Pacific Biosciences RS II system (Menlo Park, CA, USA) protocols for single-molecule real-time (SMRT) circular consensus sequencing (CCS) of MHC class I full-length transcript amplicons were followed as previously described (Karl et al. 2017; Westbrook et al. 2015). The 24 samples selected for sequencing were processed in three separate pools of eight samples each, with a mixture of two and three different 5′ and 3′ primers, respectively (Suppl. Table 1). Each primer was tagged at the 5′ end with a unique 16-bp barcode (www.pacb.com) to allow identification of samples. For PCR amplification of differentially barcoded MHC class I samples, the following conditions were applied: 40 μl of reaction mix containing 4 μl of cDNA, 1× Phusion HF buffer, 0.2 μM dNTPs, 0.02 U/μl Phusion HS II HF Polymerase, 0.5 μM each of forward and reverse primer mixes (Suppl. Table 1). Cycling conditions were 1 min at 98 °C hot-start, followed by 22 cycles of 5 s at 98 °C, 10 s at 60 °C and 20 s at 72 °C, with a final extension of 5 min at 72 °C and 30 s at 4 °C. PCR products of ~ 1200 bp were selected by gel electrophoresis, excised and purified with a GeneJet Gel Extraction Kit (Thermo Fisher Scientific, Leon-Rot, Germany). PCRs were repeated until a total of ~ 3 μg of DNA was obtained. The amplicons were then pooled and purified twice using AMPure XP beads (Beckman-Coulter, Woerden, The Netherlands) at a 1:1 bead to DNA volume ratio. The DNA concentration of the purified samples was measured using the Qubit dsDNA HS assay kit and Qubit 2.0 Fluorometer (Therma Fisher Scientific, Waltham, Ma, USA) and should be >1 μg.

The amplicons were then sent to the Leiden Genome Technology Centre (LGTC), where the creation of the SMRTbell templates and sequencing was performed on a PacBio RSII instrument. Briefly, DNA damage and end-repair protocols were run on the pools of differentially barcoded MHC class I amplicons, adapters were blunt-ligated to the ends of the amplicons to create SMRTbell templates, unligated products were removed via exonuclease treatment, the SMRTbell templates were purified to remove adapter dimers and small products, sequencing primers and polymerase were bound to the PacBio adapters, templates were loaded onto the PacBio RS II instrument, and circular consensus sequences (CCS) were obtained by reading around each SMRTbell template multiple times (Karl et al. 2017).

Each pool was sequenced on one SMRT cell, and 465 reads were obtained on average per sample. One pool was additionally sequenced on a PacBio Sequel instrument.

### PacBio full-length amplicon analysis

At LGTC, the reads of insert files for all SMRT cells for a particular pool of samples were first combined, and all reads of insert were pre-processed to remove zero-length reads and to append the barcode name or sample ID to each read header for easier downstream tracking. Reads of 900–1200 bp were extracted from the pre-processed reads of insert files (Karl et al. 2017) (www.pacb.com).

Fastq files were imported into Geneious Pro (version 9.0) (Biomatters Limited, Auckland, New Zealand) (Kearse et al. 2012). Sequences were then mapped against a database containing full-length or partial *Paan-AB* transcripts at 100% identity to pull out all reads of known partial or full-length sequences, leaving only putative novel or extensions of known transcripts for downstream analysis. Identical sequences were then clustered, and only clusters supported by at least three independent reads were retained. Reads of the individual clusters were used to pull out all extensions of known sequences; these extensions were retained for validation alongside the putative novel transcripts. A fasta file of putative novel transcripts and a genotyping table with read counts per sample were generated from the final mapping step.

Putative novel sequences and extensions of known transcripts were manually validated using Geneious Pro (version 9.0) (Karl et al. 2017). All novel and extension sequences supported by three or more perfectly identical full-length reads were submitted to the NCBI GenBank for accession numbers, and to the Immuno Polymorphism Database for the Major Histocompatibility Complex genes of Non-Human Primates (IPD-MHC NHP) (http://www.ebi.ac.uk/ipd/mhc/nhp/index.html) for official nomenclature (de Groot et al. 2012; Maccari et al. 2017) (Suppl. Table 2).

### Phylogenetic analysis

The evolutionary history of the *Paan-A* alleles was inferred by using the maximum likelihood method based on the Jukes–Cantor model. The tree with the highest log likelihood (− 6824.3553) is shown. The percentage of trees in which the associated taxa clustered together is shown next to the branches. Initial tree(s) for the heuristic search were obtained automatically by applying neighbor-joining and BioNJ algorithms to a matrix of pairwise distances estimated using the maximum composite likelihood (MCL) approach and then selecting the topology with superior log likelihood value. The tree is drawn to scale, with branch lengths measured in the number of substitutions per site. The analysis involved 38 nucleotide sequences. Codon positions included were first + second + third + noncoding. There was a total of 1098 positions in the final dataset. Evolutionary analyses were conducted in MEGA7.

The evolutionary history of the *Paan-B* alleles was inferred using the neighbour-joining method. The optimal tree with the sum of branch length = 1.85719168 is shown. The tree is drawn to scale, with branch lengths in the same units as those of the evolutionary distances used to infer the phylogenetic tree. The evolutionary distances were computed using the Nei-Gojobori method and are in the units of the number of synonymous substitutions per synonymous site. The analysis involved 130 nucleotide sequences, and all ambiguous positions were removed for each sequence pair. There was a total of 364 positions in the final dataset. Evolutionary analyses were conducted in MEGA7.

## Results

### Paan-A STR screening

Microsatellite typing with the A region-specific markers, D6S2854 and D6S2859, which allowed succesful polymorphism screening and haplotyping in various Old World monkey species (Doxiadis et al. 2011), was also conducted on the samples of the olive baboon cohort (*N* = 154). As expected, screening with both microsatellites revealed a high degree of length and copy number variation in olive baboons, especially for D6S2854. This cohort also comprises related animals, and particular combinations of STR-length patterns were found to segregate together in the olive baboon families, illustrating that haplotypes can be deduced. In such a way, 23 different haplotypes were able to be defined with one to four D6S2854 and zero to two amplicons for D6S2859 per haplotype (Table 1).

**Table 1:**
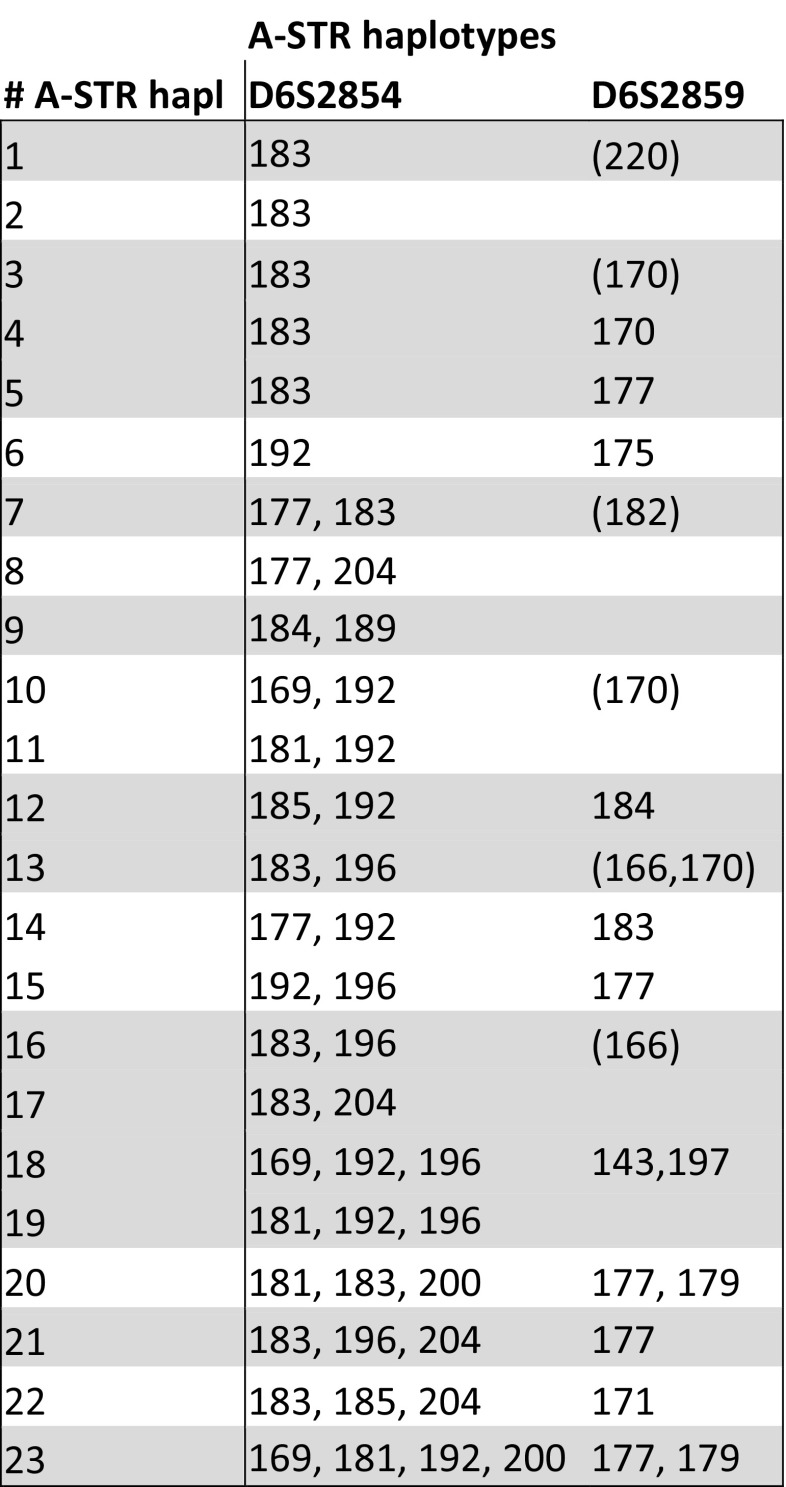
A-STR haplotypes

*Paan-A* hapl defined shaded in grey. () mostly, but not always detected

### Paan-A and Paan-B allele discoveries

Since the results of the STR prescreening suggested high levels of diversity with regard to copy number variation and polymorphism within the *Paan*-*A* region, we decided to perform a thorough characterisation of the *Paan* class I region. Therefore, MHC class I transcripts of a selected panel of 24 cohort animals were analysed using two NGS platforms. In the first instance, cDNA of class I exon 2–exon 3 was amplified and sequenced on a Roche 454 platform with long-read software, using specially developed primer sets and two subsequent PCR reactions. Because Roche stopped the production of NGS kits, allele discovery has been continued by way of full-length *Mhc* class I *Paan*-*A* and *B* cDNA sequencing on PacBio instruments by using generic primer sets developed originally for macaques (Karl et al. 2017) (Suppl. Table 1). However, both methods detected more or less the same number and combination of alleles with a comparable number of reads. The discrepancies observed are likely due to primer inconsistencies. Combining the results obtained by the two NGS platforms resulted in the discovery of a total of 21 and 80 *Paan-A* and *Paan-B* full-length sequences, respectively. Of these, 18 and 60 represent unreported *Paan-A* and *Paan-B* alleles or reflect extensions to known sequences (Suppl. Table 2).

**Fig. 1:**
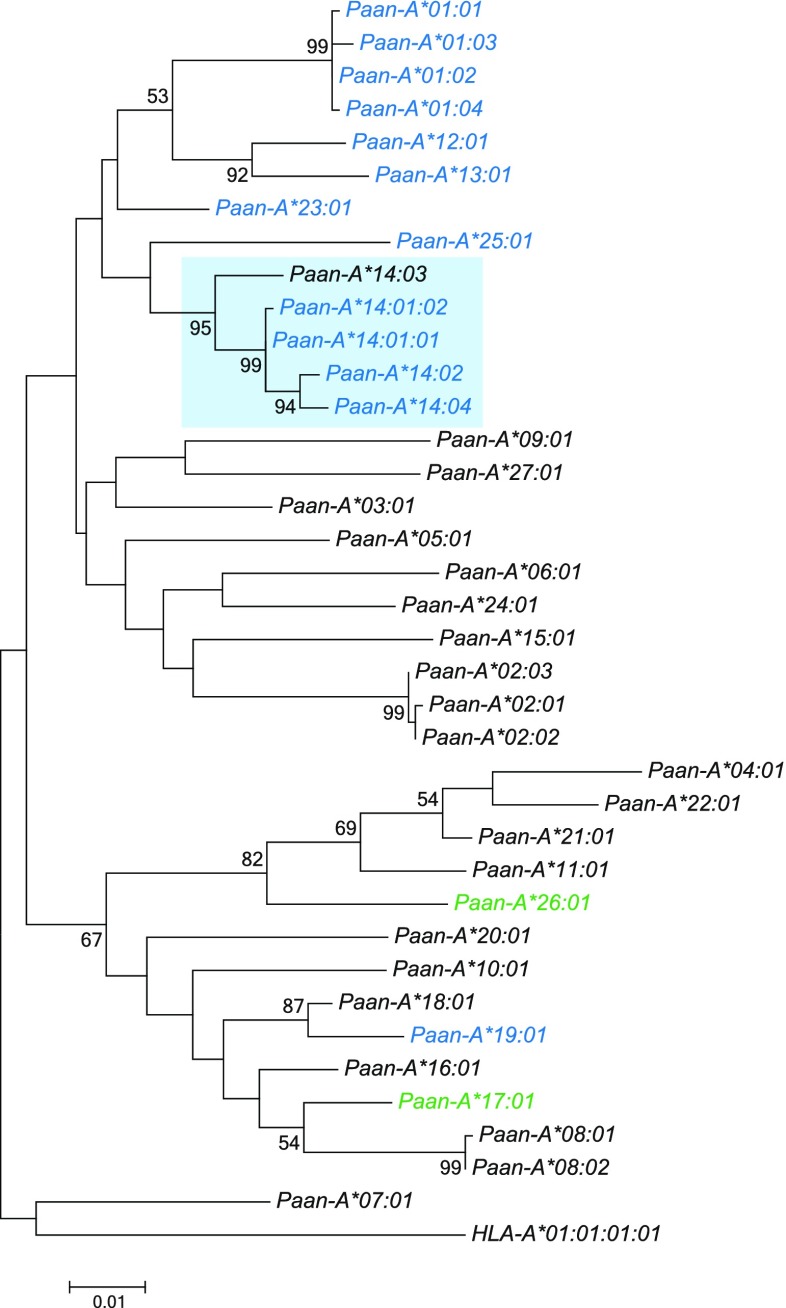
Phylogenetic tree of *Paan-A* transcripts. The evolutionary history was inferred by using the maximum likelihood method based on the Jukes–Cantor model. Evolutionary analyses were conducted in MEGA7. The Paan-A*14 alleles are indicated by a *blue* background, and the alleles with a canonical Bw6 motif are indicated in *blue*, whereas *Paan-A* alleles with an identical Bw6 motif are shown in *green*

In general, one to four highly transcribed *A* alleles (majors) are present per animal. Based on phylogenetic analyses, a total of 18 different *A* lineages were able to be defined (Fig. 1). As can be seen, most lineages are separated by deep branch lengths, indicating the large genetic distances that exist between them. In most cases, lineages appear to display extremely low levels of polymorphism or no evidence of polymorphism at all. Five alleles belonging to the A*14 lineage (Fig. 1) are all minors characterised by low transcription levels.

**Fig. 2:**
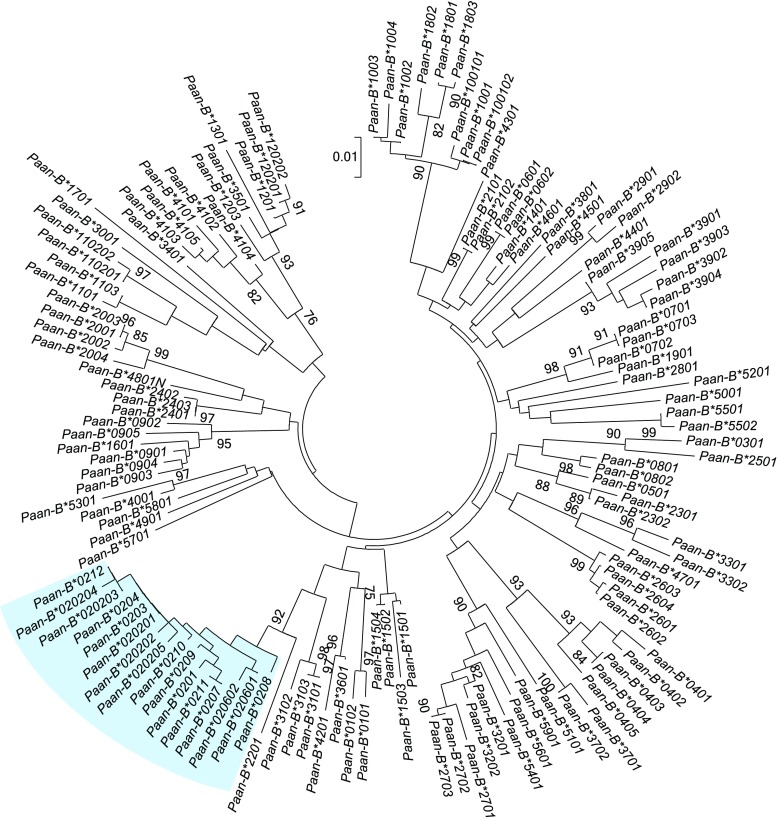
Phylogenetic tree of *Paan-B* transcripts. The evolutionary history was inferred using the neighbour-joining method. The optimal tree with the sum of branch length = 1.85719168 is shown. The percentages of replicate trees in which the associated taxa clustered together in the bootstrap test (1000 replicates) are shown next to the branches. Evolutionary analyses were conducted in MEGA7. The Paan-B*02 alleles are indicated by a *blue* background. Allele names are written without colon, e.g. Paan-B*0212 instead of the official allele designation Paan-B*02:12

Per animal, eight to 13 bona fide *B* transcripts have been detected (Suppl. Table 3). Four related animals (V9911D, V933BB, V941EA, and AA833C; Suppl. Table 3) show two additional transcripts that most likely represent pseudogenes, as nucleotide deletions lead to the disturbance of the reading frame. These have been named B*48:01ps and *B ps2*. Phylogenetic analysis demonstrated that *Paan-B* alleles cluster into a high number of different lineages, with deep branch lengths, and show differential transcription levels (Fig. 2; Suppl. Table 3). One of the lineages, Paan-B*02, is polymorphic, and its alleles form a separate branch in the phylogenetic tree (Fig. 2). Most *Paan*-B*02 alleles seem to be majors, which are most likely encoded by a separate gene, as some of the animals are heterozygous. Some animals, however, seem to lack a B*02 gene/allele, suggesting that it is not fixed at the haplotype level, whereas other animals may have a duplicated set, as more than one allele is detected per haplotype. In macaques, a more or less similar situation is observed for the *I* locus, which maps in the *B* region, and is fixed, since one copy of an *I* allele is present on each haplotype (Urvater et al. 2000). To some extent, other lineages such as B*39 and B*04 also show allelic variations as well, whereas for most lineages, no allelic polymorphism is observed.

### Definition of Paan-AB haplotypes

**Fig. 3:**
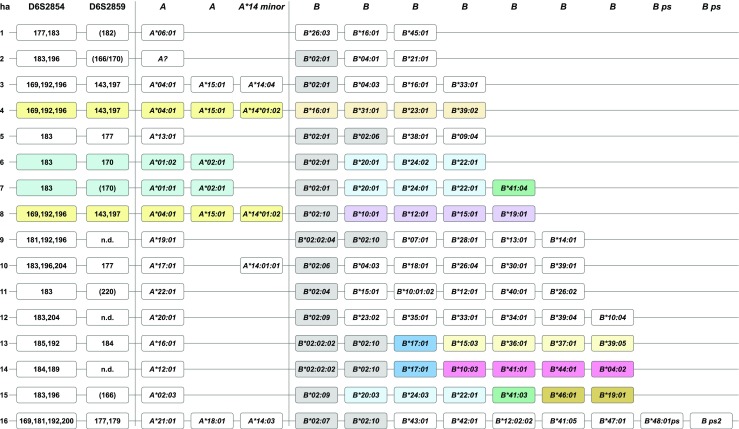
*Paan-AB* haplotypes defined by A-STR typing and NGS sequencing. B*02 alleles are given in bold. Possible crossing-over events between the *A* and *B* region or within the *B* region are illustrated by different colours. The D6S2859 lengths in brackets indicate that they are detected in most but not all animals within the respective haplotype; n.d. equals not detected; A? indicates that no *A* allele could be defined for this haplotype

Based on co-segregation of *A* and *B* genes/alleles in related animals, we were able to deduce *Paan-AB* haplotypes (Fig. 3). Most *Paan-A* transcripts could be assigned to haplotypes and are confirmed by the A-STR haplotypes described above (Table 1, grey background). Likewise, nearly all *B* genes/alleles could be assigned to haplotypes (Suppl. Table 3, colour-coded). In such a way, it was possible to define 16 *Paan-AB* haplotypes, which consist generally of one to three *A* and three to seven *B* transcripts (Fig. 3). With the primer combinations used, however, no *A* transcript has been amplified that can be allocated to haplotype 2, which is present in four *Paan-AB* heterozygous animals, (Suppl. Table 3; Fig. 3, ha 2). This indicates that some haplotypes may lack a functional *A* gene, although we cannot exclude the possibility that some transcripts are not amplified with the current set of primers. If two *A* genes are present on the same haplotype—for instance, Paan-A*04:01 and -A*15:01—both can be majors, and a minor A*14 allele may additionally be observed (Fig. 3, ha 3, 4 and 8).

The situation is more complex for *B* genes/alleles; however, as on nearly every haplotype, major *B* transcripts are detected together with multiple *B* genes with lower transcription levels. These results are comparable to macaques, in which a *B* region comprises several major *B* genes together with up to seven minor transcripts (Karl et al. 2013, 2017; Otting et al. 2005). In addition to seven bona fide *B* transcripts, haplotype 16, which is present in four animals (Suppl. Table 3), contains two pseudogenes.

Although most of the haplotypes consist of different *A* and *B* genes, the combinations A*04:01, A*15:01, A*14:01:02/14:04 and A*01:02 and A*02:01 are present on three or two haplotypes, respectively. Furthermore, the B*02 lineage alleles are exceptional; *Paan*-B*02:01, for example, is present on five of the 16 haplotypes, in combination with different other *B* genes (Fig. 3).

### Paan-AB haplotypes generated by recombination-like processes

In Indian rhesus macaques, it has been shown that the reshuffling of *A, B*, and *DR* region segments by recombination-like events can generate ‘new’ haplotypes that accumulated over long evolutionary time spans (Doxiadis et al. 2013). Here, we also observed haplotypes (e.g. Fig. 3, ha 4, and 8) that seem to have been generated in the past by recombination-like events between the *A* and *B* regions. In contrast to Indian rhesus macaques, however, crossing-over events map also *between B* region loci; for instance, haplotypes 6 and 7 (Fig. 3) are nearly identical apart from an additional gene, B*41:04, which is present on haplotype 7. Another example is highlighted by haplotypes 13 and 14, where three of the seven *B* genes—namely, B*02:02:02, B*02:10 and B*17:01—are shared between both haplotypes, whereas the other four *B* genes are different. An additional illustration of breakpoints within the *B* region is provided by haplotypes 7 and 15. The observations of such ‘patchwork haplotypes’ suggest that recombination-like processes have been the reason for their emergence. In addition, gene conversion events may have switched certain genes as for example B*04:03 from one haplotype to the other, as such contributing to the emergence of new haplotypes.

### Distribution of Bw4 and Bw6 motifs

Bw4 and Bw6 are mutually exclusive epitopes on the alpha 1 domain of HLA class I molecules (pos 77–83), Bw4 of which binds an array of diverse allotypes of immunoglobulin-like receptors of NK cells (KIR) in humans (Cella et al. 1994; Gumperz et al. 1995, 1997; Lutz 2014). Since Bw4 and Bw6 epitopes are also known to be present on class I molecules in macaques, we searched for their presence/absence on class I molecules in the olive baboon. As in macaques, no identical Bw4 motifs (NLRIALR) can be observed in Paan-A or Paan-B molecules. Identical Bw6 (SLRNLRG) motifs, however, are present in two A allotypes: namely, Paan-A*17:01 and Paan-A*26:01, and in the Paan-B*18 lineage members, as well as Paan-B*42:01 and Paan-B*54:01 (Suppl. Table 4). Furthermore, the canonical Bw6 motif (NLRNLRG), which is present in Mamu*-*A1*002:01, and responsible for binding to KIR3DL05 (Colantonio et al. 2011; Rosner et al. 2011), is also defined in several Paan-A allotypes, namely, those of the Paan-A*01 and Paan-A*14 lineage (except Paan-A*14:03), in addition to Paan-A*19:01, *23:01, and *25:01 (Suppl. Table 4)*.* The nucleotide sequences of all of them, with the exception of Paan-A*19:01, form a seperate branch in the phylogenetic tree (Fig. 1).

## Discussion

Long-range NGS on two different platforms, Roche 454 and PacBio SMRT, allowed the discovery of a high number of MHC class I *A* (*n* = 21) and *B* alleles (*n* = 80) in the olive baboon. For *Paan-A* and *Paan-B* alleles, different transcription levels have been observed, and it is a phenomenon known for other OWM species such as rhesus, cynomolgus, and pigtailed macaques (Fernandez et al. 2011; Karl et al. 2013, 2017; Lian et al. 2016; Otting et al. 2005, 2007, 2009, 2012). Additionally, the occurrence of different numbers of loci per haplotype—especially within the *B* region—is also comparable to the situation in macaques. Thus, in olive baboons as in other OWM, the diversity of the class I region is caused mainly by CNV and the different content of the respective genes, and not, as in humans, by allelic variation of the *A* and *B* genes. A peculiarity of olive baboons, however, appears to be the frequent occurrence of two different major *A* transcripts per haplotype, a phenomenon that is less often observed in macaques.

Indian rhesus macaque haplotypes appear to be the result of recombination-like processes between MHC regions such as *Mamu*-*A* and *B* (Doxiadis et al. 2013). Recombinations *within* one region like the *B* region, however, which may be the reason for such patchwork haplotypes, have not often been observed when using Sanger sequencing (de Groot et al. 2014; Doxiadis et al. 2013). These are more easily visible with high resolution NGS techniques. The existence of patchwork haplotypes within the class I *B* region is more often observed in certain macaque species such as cynomolgus macaques. Additionally, the origin of the monkeys seems to play a role, since patchwork hapotypes are more frequent in Chinese rhesus macaques than in rhesus macaques of Indian origin (Karl et al. 2013, 2017). Differential recombination dynamics have been characterised for various macaques species (de Groot et al. 2014). Thus, it appears plausible that the frequent observation of such patchwork haplotypes within the *Paan*-*B* region is a peculiarity of olive baboons.

Patchwork haplotypes are described further within the *DRB* region of cynomolgus but not rhesus macaques (Doxiadis et al. 2010). In cynomolgus macaque species, the duplication of loci, especially of *DRB6* pseudogenes, is often detected, and it has been discussed as to whether these pseudogenes may play a prominent role in the recombination process due to the presence of retroviral elements that may promote recombination (Doxiadis et al. 2008; Kulski et al. 1999). Similarly, in olive baboons, the Paan-B*02 lineage/locus is sometimes duplicated, which may indicate that the locus itself or surrounding DNA segments are prone to chromosomal breaks. The MHC region of OWM and Hominoids has been subjected to expansions and contractions. The MHC of macaques in particular has been thoroughly studied, and several rounds of duplications have been shown for their class I region, caused mainly by the incorporation of transposable elements (Dawkins et al. 1999; Kulski et al. 1999). Within the *Mamu-B* region, specific endogenous retroviral sequences have been described that lead to the duplication of certain *B* loci and sometimes subsequently to the truncation of the adjacent genes (Doxiadis et al. 2009). Comparable results are gained by the analysis of the *A* region in macaques (Doxiadis et al. 2011; Kulski et al. 1999). Thus, it appears to be plausible that transposable elements also exist within the *B* region in baboons, which may initiate breaks and thus cause recombination-like processes and duplications. Further research is needed to determine the presence of such sequences, which promote expansion and recombination.

Since OWM possess, in contrast to humans, multiple *A* and *B* transcripts per haplotype, it may be assumed that some of their products will have a specialised function. Such a specialist MHC class I molecule, which seems to be transported to the cell surface only when suitable peptides become available, has been described recently for the rhesus macaque (de Groot et al. 2017b). In addition to their peptide presenting role, class I molecules of human and non-human primates serve as ligands for NK cell receptores as KIR or CD94/NKG2. In human immunodeficiency virus infections (HIV) survival is highly linked to host KIR and HLA class I genotypes (Alter et al. 2007). However, for macaques and other OWM, which, for instance, serve as model species for the simian immunodeficiency virus-induced AIDS, MHC-KIR interactions are not fully understood. In contrast to humans but as in rhesus macaques, no completely identical Bw4 epitopes can be detected for class I A (Fig. 1) and B molecules in the olive baboon, though Bw6 epitopes can be (Suppl. Table 4). However, binding assays between selected class I molecules or tetramers, respectively, have shown KIR binding to macaque class I A molecules (Colantonio et al. 2011; Maloveste et al. 2012; Rosner et al. 2011). Additionally, one commonly expressed molecule, Mamu-KIR3DL01, revealed similarity in canonical Bw4 epitope recognition with human KIR3DL1 (Schafer et al. 2014). In contrast to humans, molecules such as Mamu-A1*002:01, which show a canonical Bw6 epitope, have been identified as ligands for KIR3DL05 in rhesus macaques (Colantonio et al. 2011). Several class I A molecules with this canonical Bw6 motif have been determined in the olive baboon as well, including the minor Paan-A*14 lineage members, which may indicate that these molecules have a specialised function (Fig. 1). Research on KIR-MHC interactions in pig-tailed macaques has shown that a specific KIR3DL molecule recognised a broad range of MHC class I molecules with Bw4 and Bw6 but also non-Bw4 or non-Bw6 motifs (Maloveste et al. 2012). This MHC reactivity seems to be degenerative but peptide dependent. Future binding experiments between MHC and KIR molecules in baboons or other OWM species are needed to elucidate their molecular interactions.

## Electronic supplementary material

Suppl. Table 1 (XLSX 9 kb)

Suppl. Table 2 (XLSX 49 kb)

Suppl. Table 3 (XLSX 120 kb)

Suppl. Table 4 (XLSX 63 kb)

## References

[CR1] Alter G, Martin MP, Teigen N, Carr WH, Suscovich TJ, Schneidewind A, Streeck H, Waring M, Meier A, Brander C, Lifson JD, Allen TM, Carrington M, Altfeld M (2007). Differential natural killer cell-mediated inhibition of HIV-1 replication based on KIR/HLA subtypes. J Exp Med.

[CR2] Budde ML, Wiseman RW, Karl JA, Hanczaruk B, Simen BB, O’Connor DH (2010). Characterization of Mauritian cynomolgus macaque major histocompatibility complex class I haplotypes by high-resolution pyrosequencing. Immunogenetics.

[CR3] Cella M, Longo A, Ferrara GB, Strominger JL, Colonna M (1994). NK3-specific natural killer cells are selectively inhibited by Bw4-positive HLA alleles with isoleucine 80. J Exp Med.

[CR4] Colantonio AD, Bimber BN, Neidermyer WJ, Reeves RK, Alter G, Altfeld M, Johnson RP, Carrington M, O’Connor DH, Evans DT (2011). KIR polymorphisms modulate peptide-dependent binding to an MHC class I ligand with a Bw6 motif. PLoS Pathog.

[CR5] Dawkins R, Leelayuwat C, Gaudieri S, Tay G, Hui J, Cattley S, Martinez P, Kulski J (1999). Genomics of the major histocompatibility complex: haplotypes, duplication, retroviruses and disease. Immunol Rev.

[CR6] Doxiadis GG, de Groot N, Bontrop RE (2008). Impact of endogenous intronic retroviruses on major histocompatibility complex class II diversity and stability. J Virol.

[CR7] Doxiadis GG, Heijmans CM, Bonhomme M, Otting N, Crouau-Roy B, Bontrop RE (2009). Compound evolutionary history of the rhesus macaque MHC class I B region revealed by microsatellite analysis and localization of retroviral sequences. PLoS One.

[CR8] Doxiadis GG, de Groot N, de Groot NG, Rotmans G, de Vos-Rouweler AJ, Bontrop RE (2010). Extensive DRB region diversity in cynomolgus macaques: recombination as a driving force. Immunogenetics.

[CR9] Doxiadis GG, de Groot N, Otting N, Blokhuis JH, Bontrop RE (2011). Genomic plasticity of the MHC class I A region in rhesus macaques: extensive haplotype diversity at the population level as revealed by microsatellites. Immunogenetics.

[CR10] Doxiadis GG, de Groot N, Otting N, de Vos-Rouweler AJ, Bolijn MJ, Heijmans CM, de Groot NG, van der Wiel MK, Remarque EJ, Vangenot C, Nunes JM, Sanchez-Mazas A, Bontrop RE (2013). Haplotype diversity generated by ancient recombination-like events in the MHC of Indian rhesus macaques. Immunogenetics.

[CR11] Fernandez CS, Reece JC, Saepuloh U, De Rose R, Ishkandriati D, O’Connor DH, Wiseman RW, Kent SJ (2011). Screening and confirmatory testing of MHC class I alleles in pig-tailed macaques. Immunogenetics.

[CR12] de Groot NG, Otting N, Robinson J, Blancher A, Lafont BA, Marsh SG, O’Connor DH, Shiina T, Walter L, Watkins DI, Bontrop RE (2012). Nomenclature report on the major histocompatibility complex genes and alleles of Great Ape, Old and New World monkey species. Immunogenetics.

[CR13] de Groot N, Doxiadis GG, Otting N, de Vos-Rouweler AJ, Bontrop RE (2014). Differential recombination dynamics within the MHC of macaque species. Immunogenetics.

[CR14] de Groot N, Stanbury K, de Vos-Rouweler AJ, de Groot NG, Poirier N, Blancho G, de Luna C, Doxiadis GG, Bontrop RE (2017). A quick and robust MHC typing method for free-ranging and captive primate species. Immunogenetics.

[CR15] de Groot NG, Heijmans CMC, de Ru AH, Janssen GMC, Drijfhout JW, Otting N, Vangenot C, Doxiadis GGM, Koning F, van Veelen PA, Bontrop RE (2017). A specialist macaque MHC class I molecule with HLA-B*27-like peptide-binding characteristics. J Immunol.

[CR16] Gumperz JE, Litwin V, Phillips JH, Lanier LL, Parham P (1995). The Bw4 public epitope of HLA-B molecules confers reactivity with natural killer cell clones that express NKB1, a putative HLA receptor. J Exp Med.

[CR17] Gumperz JE, Barber LD, Valiante NM, Percival L, Phillips JH, Lanier LL, Parham P (1997). Conserved and variable residues within the Bw4 motif of HLA-B make separable contributions to recognition by the NKB1 killer cell-inhibitory receptor. J Immunol.

[CR18] Karl JA, Bohn PS, Wiseman RW, Nimityongskul FA, Lank SM, Starrett GJ, O’Connor DH (2013). Major histocompatibility complex class I haplotype diversity in Chinese rhesus macaques. G3 (Bethesda).

[CR19] Karl JA, Graham ME, Wiseman RW, Heimbruch KE, Gieger SM, Doxiadis GG, Bontrop RE, O’Connor DH (2017). Major histocompatibility complex haplotyping and long-amplicon allele discovery in cynomolgus macaques from Chinese breeding facilities. Immunogenetics.

[CR20] Kearse M, Moir R, Wilson A, Stones-Havas S, Cheung M, Sturrock S, Buxton S, Cooper A, Markowitz S, Duran C, Thierer T, Ashton B, Meintjes P, Drummond A (2012). Geneious basic: an integrated and extendable desktop software platform for the organization and analysis of sequence data. Bioinformatics.

[CR21] Kulski JK, Gaudieri S, Martin A, Dawkins RL (1999). Coevolution of PERB11 (MIC) and HLA class I genes with HERV-16 and retroelements by extended genomic duplication. J Mol Evol.

[CR22] Le Bas-Bernardet S, Tillou X, Branchereau J, Dilek N, Poirier N, Chatelais M, Charreau B, Minault D, Hervouet J, Renaudin K, Crossan C, Scobie L, Takeuchi Y, Diswall M, Breimer ME, Klar N, Daha MR, Simioni P, Robson SC, Nottle MB (2015). Bortezomib, C1-inhibitor and plasma exchange do not prolong the survival of multi-transgenic GalT-KO pig kidney xenografts in baboons. Am J Transplant.

[CR23] Lian XD, Zhang XH, Dai ZX, Zheng YT (2016). Cloning, sequencing, and polymorphism analysis of novel classical MHC class I alleles in northern pig-tailed macaques (Macaca leonina). Immunogenetics.

[CR24] Lutz CT (2014). Human leukocyte antigen Bw4 and Bw6 epitopes recognized by antibodies and natural killer cells. Curr Opin Organ Transplant.

[CR25] Maccari G, Robinson J, Ballingall K, Guethlein LA, Grimholt U, Kaufman J, Ho CS, de Groot NG, Flicek P, Bontrop RE, Hammond JA, Marsh SG (2017). IPD-MHC 2.0: an improved inter-species database for the study of the major histocompatibility complex. Nucleic Acids Res.

[CR26] Maloveste SM, Chen D, Gostick E, Vivian JP, Plishka RJ, Iyengar R, Kruthers RL, Buckler-White A, Brooks AG, Rossjohn J, Price DA, Lafont BA (2012). Degenerate recognition of MHC class I molecules with Bw4 and Bw6 motifs by a killer cell Ig-like receptor 3DL expressed by macaque NK cells. J Immunol.

[CR27] Naruse TK, Chen Z, Yanagida R, Yamashita T, Saito Y, Mori K, Akari H, Yasutomi Y, Miyazawa M, Matano T, Kimura A (2010). Diversity of MHC class I genes in Burmese-origin rhesus macaques. Immunogenetics.

[CR28] O’Leary CE, Wiseman RW, Karl JA, Bimber BN, Lank SM, Tuscher JJ, O’Connor DH (2009). Identification of novel MHC class I sequences in pig-tailed macaques by amplicon pyrosequencing and full-length cDNA cloning and sequencing. Immunogenetics.

[CR29] Otting N, Heijmans CM, Noort RC, de Groot NG, Doxiadis GG, van Rood JJ, Watkins DI, Bontrop RE (2005). Unparalleled complexity of the MHC class I region in rhesus macaques. Proc Natl Acad Sci U S A.

[CR30] Otting N, de Vos-Rouweler AJ, Heijmans CM, de Groot NG, Doxiadis GG, Bontrop RE (2007). MHC class I A region diversity and polymorphism in macaque species. Immunogenetics.

[CR31] Otting N, Doxiadis GG, Bontrop RE (2009). Definition of Mafa-A and -B haplotypes in pedigreed cynomolgus macaques (Macaca fascicularis). Immunogenetics.

[CR32] Otting N, de Groot N, de Vos-Rouweler AJ, Louwerse A, Doxiadis GG, Bontrop RE (2012). Multilocus definition of MHC haplotypes in pedigreed cynomolgus macaques (Macaca fascicularis). Immunogenetics.

[CR33] Otting N, van der Wiel MK, Doxiadis GG, Bontrop RE (2016). Fifty-one full-length major histocompatibility complex class II alleles in the olive baboon (Papio anubis). HLA.

[CR34] Poirier N, Mary C, Le Bas-Bernardet S, Daguin V, Belarif L, Chevalier M, Hervouet J, Minault D, Ville S, Charpy V, Blancho G, Vanhove B (2014). Advantages of Papio anubis for preclinical testing of immunotoxicity of candidate therapeutic antagonist antibodies targeting CD28. MAbs.

[CR35] Poirier N, Dilek N, Mary C, Ville S, Coulon F, Branchereau J, Tillou X, Charpy V, Pengam S, Nerriere-Daguin V, Hervouet J, Minault D, Le Bas-Bernardet S, Renaudin K, Vanhove B, Blancho G (2015). FR104, an antagonist anti-CD28 monovalent fab’ antibody, prevents alloimmunization and allows calcineurin inhibitor minimization in nonhuman primate renal allograft. Am J Transplant.

[CR36] Rosner C, Kruse PH, Hermes M, Otto N, Walter L (2011). Rhesus macaque inhibitory and activating KIR3D interact with Mamu-A-encoded ligands. J Immunol.

[CR37] Saito Y, Naruse TK, Akari H, Matano T, Kimura A (2012). Diversity of MHC class I haplotypes in cynomolgus macaques. Immunogenetics.

[CR38] Schafer JL, Colantonio AD, Neidermyer WJ, Dudley DM, Connole M, O’Connor DH, Evans DT (2014). KIR3DL01 recognition of Bw4 ligands in the rhesus macaque: maintenance of Bw4 specificity since the divergence of apes and Old World monkeys. J Immunol.

[CR39] Schochl H, van Griensven M, Heitmeier S, Laux V, Kipman U, Roodt J, Bahrami S, Redl H (2017). Dual inhibition of thrombin and activated factor X attenuates disseminated intravascular coagulation and protects organ function in a baboon model of severe gram-negative sepsis. Crit Care.

[CR40] Shiina T, Yamada Y, Aarnink A, Suzuki S, Masuya A, Ito S, Ido D, Yamanaka H, Iwatani C, Tsuchiya H, Ishigaki H, Itoh Y, Ogasawara K, Kulski JK, Blancher A (2015). Discovery of novel MHC-class I alleles and haplotypes in Filipino cynomolgus macaques (Macaca fascicularis) by pyrosequencing and Sanger sequencing: Mafa-class I polymorphism. Immunogenetics.

[CR41] Tanabe T, Watanabe H, Shah JA, Sahara H, Shimizu A, Nomura S, Asfour A, Danton M, Boyd L, Dardenne Meyers A, Ekanayake-Alper DK, Sachs DH, Yamada K (2017). Role of intrinsic (graft) versus extrinsic (host) factors in the growth of transplanted organs following allogeneic and xenogeneic transplantation. Am J Transplant.

[CR42] Urvater JA, Otting N, Loehrke JH, Rudersdorf R, Slukvin II, Piekarczyk MS, Golos TG, Hughes AL, Bontrop RE, Watkins DI (2000). Mamu-I: a novel primate MHC class I B-related locus with unusually low variability. J Immunol.

[CR43] Westbrook CJ, Karl JA, Wiseman RW, Mate S, Koroleva G, Garcia K, Sanchez-Lockhart M, O’Connor DH, Palacios G (2015) No assembly required: Full-length MHC class I allele discovery by PacBio circular consensus sequencing. Hum Immunol 76(12):891–896 10.1016/j.humimm.2015.03.022 26028281

[CR44] Wiseman RW, Wojcechowskyj JA, Greene JM, Blasky AJ, Gopon T, Soma T, Friedrich TC, O’Connor SL, O’Connor DH (2007). Simian immunodeficiency virus SIVmac239 infection of major histocompatibility complex-identical cynomolgus macaques from Mauritius. J Virol.

